# The Periosteal Bone Surface is Less Mechano-Responsive than the Endocortical

**DOI:** 10.1038/srep23480

**Published:** 2016-03-23

**Authors:** Annette I. Birkhold, Hajar Razi, Georg N. Duda, Richard Weinkamer, Sara Checa, Bettina M. Willie

**Affiliations:** 1 Julius Wolff Institute, Charité - Universitätsmedizin Berlin, Berlin Germany; 2 Max Planck Institute of Colloids and Interfaces, Potsdam, Department of Biomaterials, Germany; 3 Continuum Biomechanics and Mechanobiology Research Group, Institute of Applied Mechanics, University of Stuttgart, Germany; 4 Research Centre, Shriners Hospital for Children-Canada, Department of Pediatric Surgery, McGill University, Montreal, Canada

## Abstract

Dynamic processes modify bone micro-structure to adapt to external loading and avoid mechanical failure. Age-related cortical bone loss is thought to occur because of increased endocortical resorption and reduced periosteal formation. Differences in the (re)modeling response to loading on both surfaces, however, are poorly understood. Combining *in-vivo* tibial loading, *in-vivo* micro-tomography and finite element analysis, remodeling in C57Bl/6J mice of three ages (10, 26, 78 week old) was analyzed to identify differences in mechano-responsiveness and its age-related change on the two cortical surfaces. Mechanical stimulation enhanced endocortical and periosteal formation and reduced endocortical resorption; a reduction in periosteal resorption was hardly possible since it was low, even without additional loading. Endocortically a greater mechano-responsiveness was identified, evident by a larger bone-forming surface and enhanced thickness of formed bone packets, which was not detected periosteally. Endocortical mechano-responsiveness was better conserved with age, since here adaptive response declined continuously with aging, whereas periosteally the main decay in formation response occurred already before adulthood. Higher endocortical mechano-responsiveness is not due to higher endocortical strains. Although it is clear structural adaptation varies between different bones in the skeleton, this study demonstrates that adaptation varies even at different sites within the same bone.

Bone structure and material properties have presumably adapted to meet evolutionary pressures, balancing the contradictory needs of stiffness and toughness. As a result, long bones are lightweight, tubular structures in which mass is placed at a distance from the neutral axis, thereby increasing the resistance to bending and torsion^1^. This tubular structure is defined by its outer (periosteal) and inner surfaces (endocortical and trabecular). Bone continually adapts to changing external loading conditions via (re)modeling (modeling and remodeling) processes. Modeling (spatially independent resorption and formation) and remodeling (spatially and temporally dependent resorption and formation) processes construct and reconstruct the skeleton by the removal and formation of bone packets that mediate the size, architecture, mass, and consequently the bone’s strength. Periosteal and endocortical (re)modeling influences both the cross sectional area of the bone and the mean distance between the endocortical and periosteal surfaces, i.e. the cortical thickness.

During growth in humans, periosteal apposition exceeds endocortical resorption, leading to a net bone gain and cortical thickening^2^. After completion of longitudinal growth, resorption and formation processes are assumed to be balanced. However, there is evidence that already during adulthood bone mass decreases^3^,^4^. Population-based, cross-sectional studies have shown that endocortical resorption, derived from measurements of the total medullary area expansion, increases before middle age and continues throughout life in men and women^3^. At the same time, periosteal apposition, derived from measurements of increases in bone diameter, remains constant in elderly men^5^ and decreases in postmenopausal women^6^,^7^. Although this long-term structural development of the cortex is well documented, much less clear are the reasons for this different behavior of the endocortical and periosteal surfaces. Cortical thinning could occur as a consequence of a reduction in sex steroids later in life that leads to increased endocortical resorption^3^,^8^,^9^, evidenced by increased endocortical resorption depth and activation frequency^10^,^11^. It is unclear whether bone cells at or close to the two surfaces act differently, in particular, whether they respond differently to mechanical stimulation. This study asks the question of whether the mechano-responiveness of the bone is not equal at the two surfaces and therefore, could contribute to age-related cortical thinning.

Long-term processes of cortical restructuring during aging are affected by short-term responses of the bone to mechanical loading occurring over the whole life span. It has been proposed that age-related bone loss can be at least in part attributed to a decrease in the mechano-responsiveness of the bone. Randomized control trials incorporating high-intensity, impact and resistance exercise have shown marked age-dependent outcomes^12^,^13^. It remains unclear how this loss in responsiveness to mechanical loading may contribute to age-related cortical thinning by altering formation and resorption on the endocortical and periosteal surfaces. Using animal models, it has been suggested that load-related increases in periosteal bone formation are attenuated with aging^14^,^15^,^16^,^17^, although other studies have reported no age-related difference in periosteal bone formation^18^,^19^,^20^,^21^. A recent study using controlled tibial loading in C57Bl/6 female mice reported increased periosteal and endocortical bone formation rates with loading in female C57Bl/6 mice^22^. However, using static microCT and dynamic histomorphometry, the contribution of the resorption side of (re)modeling to the structural changes was not investigated. We recently presented an approach for combined investigation of bone formation and resorption dynamics using longitudinal *in-vivo* microCT combined with an automated computer-based evaluation^23^,^24^,^25^. With this method, the spatial distribution and size of formed and resorbed bone volumes are determined by comparing consecutive images, which enables the monitoring of bone surface movements over time. In contrast to conventional histomorphometric methods, this approach allows detailed time-dependent *in-vivo* quantification of the kinetics of three-dimensional bone (re)modeling processes. Using this method, we previously showed that mechanical stimulation acts stronger on bone formation (by increasing it) than on bone resorption (by suppressing it). In particular, an overall increased mineralizing surface was identified as the main target of mechanical stimulation, rather than a greater thickness of individual formed bone packets^23^.

The aim of this study is to analyze the short-term response of the endocortical and periosteal surfaces to mechanical stimulation. While the above-mentioned age-related structural changes occur over months in mice (and years in humans), the response to mechanical simulation happens much faster within a few weeks. This ability of mechanical stimulation to move the endocortical and periosteal surfaces due to bone apposition and resorption was studied in mice of different ages. We hypothesized that the adaptive response is different on the opposing two surfaces and therefore, this site-specific effect could contribute to cortical thinning. We investigated the reaction to mechanical stimulation of cortical bone at both the endocortical and periosteal surfaces of young (10 week old), adult (26 week old), and elderly (78 week old) female C57Bl/6J mice subjected to *in-vivo* loading. While the age-specific influence of additional compressive loading on (re)modeling processes was studied in the loaded left tibia, the right tibia was exposed only to physiological loading conditions and therefore, served as a control. To support the interpretation of the obtained results, the mechanical environment at both surfaces was determined using finite element analysis.

## Methods

3D dynamic *in-vivo* morphometry, a method to quantify bone formation packages and bone resorption cavities using serial *in-vivo* CT data was developed previously for the analysis of trabecular bone^24^,^26^,^27^ and was extended to investigate cortical bone adaptation^23^,^25^. In the present study, the segmentation algorithm of this method was adapted to separately analyze endocortical and periosteal surfaces. MicroCT data from an experimental study^23^ were analyzed to monitor structural changes of the endocortical and periosteal surfaces separately, and to calculate at each surface the static and dynamic bone morphometric parameters. In the following briefly described are 1. the *in-vivo* experiment, 2. the image processing method, with the focus on the new segmentation algorithm, the data evaluation based on 3. static and 4. dynamic morphometry and 5. the FE model^28^, which was used in this study to determine strain differences between endocortical and periosteal regions.

### *In-vivo* bone adaptation experiment & longitudinal *in-vivo* μCT imaging

As previously reported^17^,^23^, the left tibiae of 29 female C57Bl/6J mice (10 weeks: n = 6, 26 weeks: n = 13, 78 week: n = 10; Jackson Laboratories, Sulzfeld, Germany) underwent *in-vivo* cyclic compressive loading of the left tibia. Loading parameters included: 216 cycles applied daily at 4 Hz, 5 days/week (M-F), for 2 weeks, delivering −11 N loads to the 10 and 26 week old mice and −9 N to the 78 week old mice, which engendered +1200 με on the medial surface of the tibial mid-shaft, determined by prior *in-vivo* strain gauging experiments. According to the loading protocol and assuming linear elasticity, maximum strain rates are 0.016 ε s^−1^. Previous histomorphometric analysis of loaded tibiae using these strain levels confirmed that bone formation occurred through lamellar bone formation with no evidence of woven bone present in any age group^17^. We have never previously observed intracortical fluorochrome labeling at the same load/strain levels used in the current study, thus suggesting a lack of microdamage. The right tibia served as an internal control. On day 0, prior to the beginning of the experiment and on day 5, 10 and 15 the tibial midshafts were imaged *in-vivo* in a microCT (VivaCT 40, Scanco, Switzerland; nominal isotropic image resolution 10.5 μm, 55 kVp, 145 μA, 600 ms integration time, no frame averaging). To prevent motion artifacts, anesthetized mice are constrained in a custom-made plastic mouse bed during imaging. Animal experiments were carried out according to the policies and procedures approved by the local legal representative (LAGeSo Berlin, G0333/09).

### Image registration, fusion & automatic segmentation

Consecutive microCT images of the same bone, acquired at different time points, were geometrically aligned in a common coordinate system. To improve the registration result, the fibula was removed from all data sets. The registration algorithm included: (1) rigidly translating the later images onto the earlier reference image to superimpose the centers of gravity and align the principal axes, (2) the image from the later time point was registered onto the reference image using a 3D rigid registration with normalized mutual information as optimization criterion, (3) a Lanczos interpolation was applied to registered images to transform them into the coordinate system of the reference data set. As a result, all four images of each bone had a common coordinate system and the same voxel size. Images were cut to 5% of tibia length.

The segmentation algorithm consisted of four main parts: (1) Thresholding to extract bone region: Images were Gaussian filtered and binarized using a global threshold of 423/1000 (813 mg HA/cm^3^), which was determined based on the grey value histogram of the whole ROI. (2) Segmentation to exclude mineralized tissue from cortical ROI: Trabecular structured mineralized tissue present in the medullary canal was automatically removed^24^. (3) A new segmentation algorithm was developed and implemented to separate cortical bone into endocortical and periosteal regions. First, the outer periosteal and the inner endocortical surface of the cortical bone compartment were automatically extracted to enable a separate analysis from the same data set. Therefore, the cortical shell was divided into an endocortical and a periosteal region using morphological image processing by applying non-linear operations related to the shape of features in the image. To close the cortical shell and remove holes, such as blood vessels, a closing filter was applied. Then, the whole structure was filled using a flood-fill operation. Subsequently, the structure was shrunk by a thickness, which was set to be three times the expected surface movement during the observation time of 15 days on the periosteal surface, derived from previous histomorphometric analysis^17^, starting from the outer surface of the cortical shell. A masking of the input image with this “shrunk image”, i.e. to calculate the intersection of both images, defines the endocortical region for the evaluation. A masking of the input image with the image part removed by shrinking, defines the periosteal region. From these two region labels the endocortical and periosteal surfaces are obtained, which are defined as the interface between the endocortical/periosteal regions and the background region. To minimize partial volume effects, we deleted one voxel from all surfaces to prevent overestimation. The thickness of the new surface voxels is set to 1.5 voxels to compensate for this. Therefore, all parameters are slightly underestimated; however, as previously shown, they correlate well with histological determined parameters of MS/BS^23^,^24^. The two regions are then evaluated separately. (4) Determining sites of newly formed, resorbed and quiescent cortical bone regions: Sites of bone formation and resorption were identified by comparing the images of the same bone in a common coordinate system at the different time points. Voxels labeled as bone in both data sets were defined as quiescent bone, whereas voxels only labeled as bone in the later measurement were considered formed, and voxels only labeled as bone in the earlier measurement correspond to resorbed bone.

### Three-dimensional static morphometry

Using the registered data sets 3D static morphometric parameters were calculated for all four time points, including total cortical bone volume (*Ct.BV*), average cortical area (*Ct.Ar* = *Ct.BV*/(*number of image slices* ∗ *slice thickness*)) and mean cortical thickness (*Ct.Th*), using a distance transform (distance to the closest boundary from each point). *Ct.Ar* and *Ct.Th* of the 10 week old mice and of a subset of the 26 week old mice have been previously analyzed in a similar region of the un-registered data sets^17^. However, when performing longitudinal image acquisitions, variation in the scan region can influence morphometric parameters, and registration of the data sets can improve the reproducibility of static measures^29^.

### Three-dimensional dynamic morphometry

*D*ynamic 3D (re)modeling morphology parameters for absolute changes (day 0 → day 5, day 0 → day 10, day 0 → day 15) normalized to values at the beginning of the experiment (day 0) were calculated for normalized newly mineralized bone volume (*MV/BV*) and normalized eroded bone volume (*EV/BV*). Normalized mineralizing surface area (*MS/BS*), mean thickness of individual formation packages (mineralization thickness, *MTh*), normalized erosion surface area (*ES/BS*) and mean depth of bone erosion cavities (*ED*) were, due to the limited resolution, determined only for the 15 days interval. If not mentioned otherwise, reported values correspond to the full observation time of 15 days. For completeness and to allow comparison with earlier measured values using histomorphometry we also report the three-dimensional volumetric bone formation (*3D BFR*) and resorption rates (*3D BRR*), and the three-dimensional mineral apposition (*3D MAR*) and resorption rates (*3D MRR*). However, it should be emphasized that all these parameters are obtained simply by dividing dynamic (re)modeling morphology parameters introduced above by the time of observation, e.g. 3D MAR defined as bone thickness in μm formed per days is calculated as the value of MTh in μm divided by 15 days. Quantitative parameters of both surfaces (endocortical and periosteal separately) were previously validated by a correlation of histological and microCT-derived MAR and MS/BS (n *=* 5 bones/group; 30 bones total) for the same imaging protocol and regions as analyzed in the current paper^23^.

### Finite element analysis

The local mechanical strains induced by applied external loading within the mice tibias were determined using finite element analyses (Abaqus, Dassault Systemes Simulia, Johnston, RI, USA). Details and validation of the FE models were reported elsewhere^28^ and are only briefly summarized here. Finite element models for analyzing the local strain environment induced within the mice tibia bones during loading have been developed for the three age groups (10 week old, 26 week old, and 78 week old). Models of the whole mouse tibiae were built based on *in vitro* microCT data at isotropic voxel resolution of 9.91 μm (Skyscan 1172, Kontich, Belgium; 100 kVp, 100 μA, 360°, using 0.3° rotation steps, 3 frames averaging). The boundary conditions were set to mimic the experimental set-up. To replicate the loading of the limbs during the experiment, −11 N were applied to bones of 10 and 26 weeks old mice and −9 N to bones of 78 weeks old mice. Using these models, the mechanical strain distribution during loading on the periosteal and endocortical surfaces of the mid-diaphysis of the bones was determined.

### Statistical analysis

The effect of loading (left loaded tibia, right control tibia), region (endocortical, periosteal) and age (10, 26, 78 week old mice) as well as interactions between terms was assessed using repeated measures ANOVAs. Differences between loaded and control limbs and between endocortical and periosteal surface were assessed by paired t-tests (IBM SPSS Statistics 19; IBM Corp; Armonk, NY, US). Values are presented as mean ± standard deviation and statistical significance was set at p < 0.05.

## Results

### Effect of age on endocortical and periosteal formation and resorption in control limbs

**Figure 1:**
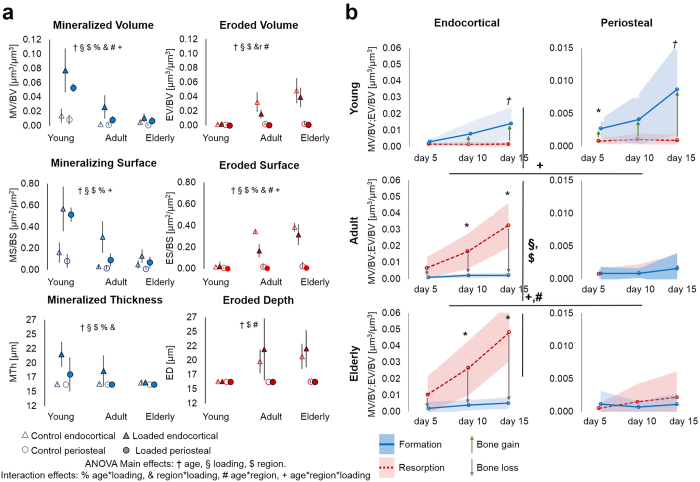
(**a**) 3D dynamic *in vivo* morphometry parameters calculated between day 0 and day 15; significance: p < 0.05. (**b**) Changes and interplay of bone formation (MV/BV) and resorption volumes (EV/BV) over 15 days in control tibiae from young, adult and elderly mice. Shaded regions correspond to standard deviations. ^*^indicates a significant difference between formation and resorption (p<0.05); ^†^indicates p = 0.06. ^#^indicates a significant difference in formation between regions. + indicates a significant difference in resorption between regions. ^§^indicates an effect of aging on bone formation. ^$^indicates an effect of aging on bone resorption.

In the control limbs of young mice, no regional differences (endocortical vs. periosteal) were observed in either formation or resorption parameters (Fig. 1a, open symbols only). In adult and elderly mice, there was nearly no resorption on the periosteal surface, but resorption occurred endocortically (EV/BV, ES/BS, ED, 3D MRR, 3D BRR; p < 0.001). Additionally, in the elderly mice endocortical formation (MV/BV and MS/BS) was slightly higher than periosteal formation (p < 0.026). Endocortical formation and resorption (p ≤ 0.016) and periosteal formation (p ≤ 0.035) parameters were affected by aging. Newly formed volume, mineralizing surface area and thickness of individual formation patches decreased on both surfaces with aging (endocortical MV/BV: young 1.4 ± 0.9%, adult 0.2 ± 0.1%, and elderly 0.5 ± 0.3%; periosteal MV/BV: young 0.8 ± 0.7%, adult 0.2 ± 0.3%, and elderly 0.1 ± 0.1%; p ≤ 0.03). Eroded bone volume, eroded surface as well as depth of resorption cavities increased on the endocortical surface with aging (EV/BV: young 0.1 ± 0.1%, adult 3.3 ± 1.4% and elderly 4.8 ± 1.8%), whereas periosteal resorption was virtually absent in all age groups (EV/BV: young 0.1 ± 0.1%, adult 0.2 ± 0.2% and elderly 0.2 ± 0.3%).

**Figure 2:**
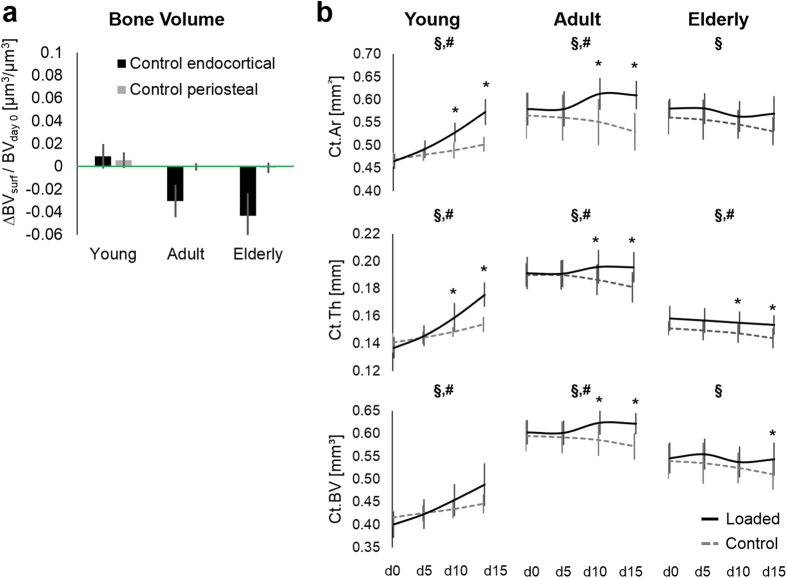
(**a**) Changes in endocortical and periosteal bone volume normalized to total bone volume (BV) of day 0 over the two weeks of the experiment (∆ BV) in the control limb (left). (**b**) Monitoring of static parameters over 2 weeks of loading. Cortical area (Ct.Ar.), thickness (Ct.Th) and total bone volume (Ct.BV) Ct.Ar. and Ct.Th. for a subset of 10 and 26 week old mice have been previously analyzed in a similar region of the unregistered data sets^17^. *Significant difference loaded vs. control. ^§^Significant difference control limbs day 0 vs. day 15. ^#^Significant difference loaded limbs day 0 vs. day 15, t-tests p < 0.05.

Looking at the net effect of both surfaces taking into account all the time intervals during the 15 days of monitoring, in the young mice a trend of higher formation than resorption was observed on the endocortical and periosteal surfaces (p ≤ 0.067; Fig. 1b top), resulting in both endocortical and periosteal expansion (Figs 1b and 2a), therefore, cortical thickening (Fig. 2b) and a subsequent gain in total bone volume (Fig. 2b bottom). In contrast, in the adult and elderly mice endocortical resorption exceeded formation (p < 0.001; Fig. 1b middle), whereas periosteal resorption and formation were balanced (Figs 1b middle and 2a), resulting in cortical thinning (Fig. 2b) and a net bone loss (Fig. 2b).

### Mechano-responsiveness of endocortical surface

**Figure 3:**
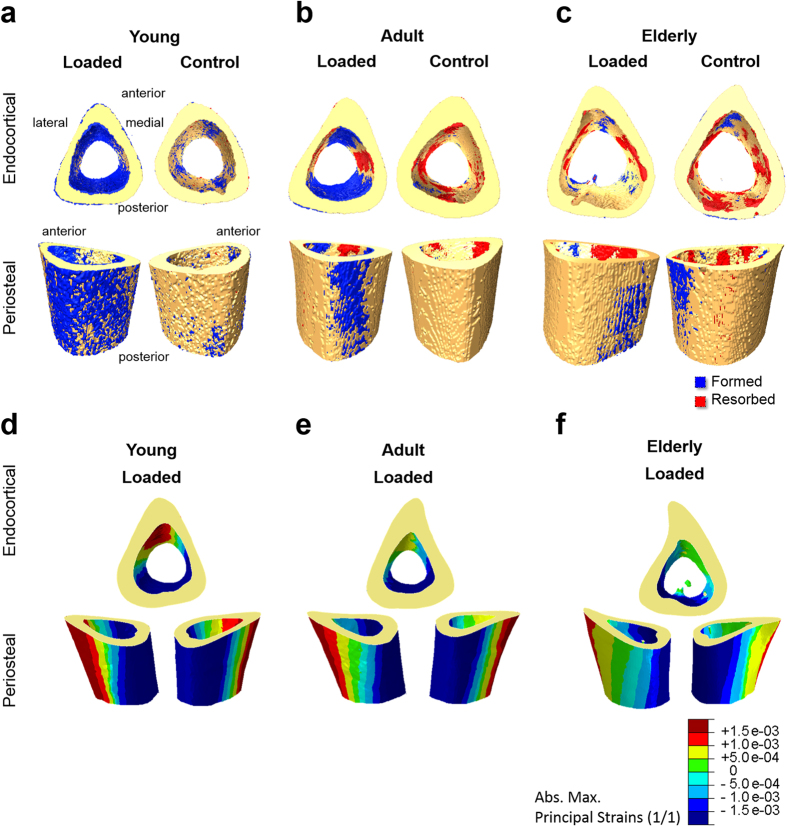
Visualization of endocortical and periosteal (re)modeling within 15 days in loaded and control young (**a**), adult (**b**) and elderly (**c**) bones. Yellow: quiescent bone; blue: newly formed bone volumes; red: resorbed bone volumes. Visualization of complete periosteal (re)modeling in supplemental videos 1,2,3,4,5,6. (**d–f**) Absolute maximum principal strains in loaded limbs of young (**d**), adult (**e**) and elderly (**f**) mice.

Controlled *in-vivo* mechanical loading of the left tibia significantly affected formation (MV/BV) via an increase in MS/BS in all three ages and an increase in MTh in young and adult mice (Fig. 1a, comparison of full and open triangles). In the tibia of young mice, loading led within 15 days endocortically to 7.7% newly formed bone (MV/BV) versus only 1.4% formed in the control limb (p ≤ 0.005; Supplementary Table S1). In the adult mice, loading led to 2.6% newly formed bone (MV/BV) compared to 0.2% in the control limb (p ≤ 0.041). In the elderly mice 1.1% newly formed bone was measured in the loaded versus 0.5% in the control limb (p = 0.038). For resorption, a significant effect of loading was only found in adult mice; EV/BV was reduced by loading to 1.6% resorbed bone volume (EV/BV) versus 3.2% in the control limb (p ≤ 0.035). Representative images of (re)modeling on the endocortical surface are given in Fig. 3 on the top. The two main features that are highly visible in the loaded cortex of adult mice are that formation and resorption occur in well-separated areas of the endocortical and periosteal surfaces, and that these regions of bone (re)modeling are extended parallel to the axis of the long bone, giving them a striped-like appearance.

### Mechano-responsiveness of periosteal surface

Loading significantly affected formation (MV/BV) via an increase in MS/BS in all three ages (Fig. 1, comparison of full and open circles), although the response was diminished with increasing age. In the young mice we found 5.3% newly formed bone (MV/BV) due to loading versus 0.9% formed in the control limb (p ≤ 0.005; Supplementary Table S2). In adult mice we measured 0.8% newly formed bone (MV/BV) due to loading versus 0.2% formed in the control (p ≤ 0.004). Elderly mice had 0.7% newly formed bone (MV/BV) due to loading versus 0.1% formed in the control. Since resorption hardly occurred already in the control animals, loading could not further reduce it and, consequently no significant effect of loading on resorption in young, adult, or elderly mice could be detected (Fig. 1a). Representative images of (re)modeling on the periosteal surface are given in Fig. 3 on the bottom and videos 1,2,3,4,5,6 showing the same features as described for the endocortical surface.

### Regional and age-related differences in mechano-responsiveness

**Figure 4:**
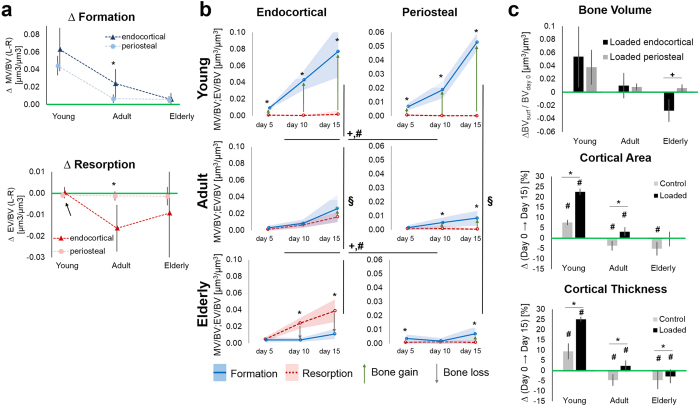
Adaptive remodeling. (**a**) Age-related changes in the effect of loading on formed (top) and resorbed (bottom) bone volume. *Significant difference between endocortical and periosteal surface. Arrow: nearly no resorption in young animals. (**b**) Changes and interplay of bone formation (MV/BV) and resorption volumes (EV/BV) over 15 days in loaded tibiae from young, adult and elderly mice. *Significant difference formation vs. resorption. ^#^Significant difference formation between regions. ^+^Significant difference in resorption between regions. ^§^Indicates an effect of aging on adaptive bone formation. Shaded regions correspond to standard deviations. (**c**) Total static changes occurring over 15 days. ^+^significant difference endocortical vs. periosteal surface. ^*^Significant difference loaded vs. control limbs. ^#^Significant difference day 0 vs. day 15.

On the endocortical surface the mechano-responsiveness of formation and resorption processes changed with aging (MV/BV, MS/BS, MTh, ES/BS, Fig. 1, full triangles, p ≤ 0.017; Fig. 4a). In contrast, on the periosteal surface only the formation response was affected by aging (MV/BV, MS/BS, MTh, Fig. 1, full circles p ≤ 0.041; Fig. 4a), as resorption was very little on this surface in all age groups. While endocortically, formation decreased significantly from young to adult and from adult to elderly mice (Fig. 4a); the mechanoresponsiveness of the periosteal surface decreased between young and adult, but showed no differences between adult and elderly mice. Although endocortical and periosteal cortical surfaces adapted to loading, there was a significantly different response to loading that occurred at the periosteal compared to the endocortical surface (MV/BV, MTh, 3D MAR, 3D BFR, EV/BV, ES/BS, 3D BRR, p ≤ 0.029) (comparison of full circles and full triangles). Furthermore, the regional response to loading was age-dependent (MS/BS, ES/BS, p ≤ 0.017). In the young mice, no adaptation differences were observed between endocortical and periosteal surfaces. In contrast, in the adult and elderly mice resorption but also formation parameters were higher at the endocortical than periosteal region (MV/BV, MS/BS, MTh, 3D MAR, 3D BFR, EV/BV, ES/BS, ED, 3D MRR, 3D BRR; p ≤ 0.045; Figs 1a and 4a).

In loaded limbs from mice of all ages, periosteal (p ≤ 0.013) formation was greater than resorption (Fig. 4b), resulting in a net bone volume gain on the periosteal surface (Fig. 4c top) and therefore periosteal expansion. On the endocortical surface of loaded bones of young mice formation exceeded resorption (p = 0.008; Fig. 4b), resulting in bone gain (Fig. 4c top); and consequently global cortical thickening (Fig. 2b solid line; Fig. 4c middle, bottom). In the adult mice, formation and resorption volumes (Fig. 4b) and the subsequent net bone volume (Fig. 4c top) were balanced on the endocortical surface. These endocortical alterations, together with the periosteal surface changes, led to global cortical thickening in the adult mice (Figs 2b and 4c). In the elderly mice, resorption exceeded formation on the endocortical surface (p = 0.001, Fig. 4b), resulting in endocortical bone loss (Fig. 4c top). Therefore, combined changes in the endocortical and periosteal surfaces resulted in global cortical thinning in elderly mice (Fig. 2b and 4c middle, bottom).

### Effect of age on strain distribution at the periosteal and endocortical surfaces

**Figure 5:**
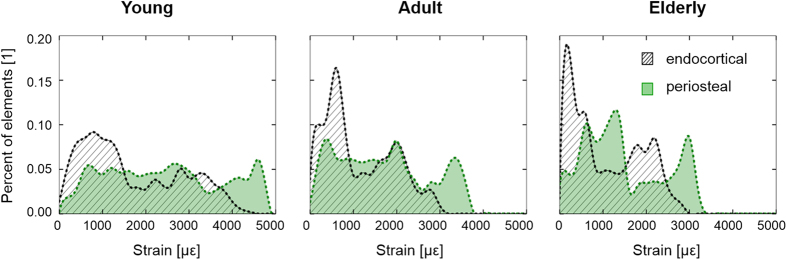
Distribution of principal strain (absolute maximum) determined by FE analysis within endocortical (hatched area) and periosteal (green area) surfaces at the tibial mid-diaphysis in a 10 (left), 26 (middle) and 78 week old mouse (right).

Within each age group, a finite element model predicted that at the loaded tibial mid-shaft the periosteal surface was under larger deformation compared with the endocortical surface. Principal strains (absolute maximum, see frequency distributions in Fig. 5) of 1790 ± 1140 με, 1240 ± 790 με and 1220 ± 800 με were determined at the endocortical surface of the tibial mid-shaft in young, adult and elderly animals, respectively. The periosteal surface at the mid-shaft exhibited 2570 ± 1330 με, 1800 ± 1030 με and 1580 ± 920 με principal strains (absolute maximum) in young, adult and elderly mice, respectively. The percent difference in principal strains between the periosteal and endocortical surfaces was lowest in elderly mice (44% in young, 45% in adult and 30% in elderly). Analysis of the distribution of mechanical strains at the two surfaces (Fig. 5) showed that most endosteal and periosteal surface points were at a similar level of strain. However in all ages, 20% of the periosteal surface points were strained at a level higher than the maximum strain detected at the endocortical surface.

## Discussion

With aging there is an increase in endocortical resorption leading to cortical thinning, however so far it remains unknown whether a change in the response to mechanical stimulation of the endocortical or periosteal surfaces occurs that might contribute to this reduction of cortical thickness. In this study, we investigated resorption and formation on the endocortical and periosteal surfaces of cortical bone in the tibia of young, adult and elderly C57Bl/6J female mice undergoing physiological loading and additional controlled *in-vivo* loading. We combined an experimental *in-vivo* approach with *in-vivo* microCT imaging and a computational evaluation procedure, which allows one to quantitatively describe both bone formation and resorption and consequently measure how the endocortical and periosteal surfaces move in response to loading. In control limbs that had only undergone physiological loading, endocortical (MV/BV, MS/BS, MTh) and periosteal (MV/BV) formation decreased with aging, while resorption (EV/BV, ES/BS, ED) increased only on the endocortical surface, resulting in a (re)modeling imbalance towards bone loss. Interestingly, the alterations in (re)modeling occurred already between young and adult mice. The largest age-related changes in the control limbs are observed in endocortical resorption surface area and depth. This finding is in accordance to earlier studies in mice^30^, and humans^5^,^6^,^31^ that used serum markers, two-dimensional formation or static structural measures to suggest that increased endocortical resorption contributed to cortical thinning.

Loading has an effect on formation at both surfaces. A closer look revealed that loading enhanced formation surface area on both the endocortical and periosteal surfaces in all three ages, although the formation response was attenuated with aging. In contrast, loading increased the thickness of individual formation packets only on the endocortical surface of young and adult mice. Studies by other groups have also reported increased periosteal^18^,^32^,^33^ and endocortical formation with loading^18^,^34^. In contrast, another study reported decreased formation on the endocortical surface with loading^35^. However, the apparent discrepancy may be due to the different mouse strain and the 2D histological method used in that investigation. Our work focuses on C57/Bl6 mice, which may be more appropriate than other strains as a model to study age-related bone loss^36^. Loading resulted in a much greater effect on the formation surface area than on the formation thickness at the periosteal surface. This holds also for the endocortical surface, although here we also observe a contribution of probably faster or longer working osteoblasts, which, leads to thicker newly formed bone packets. The greater response to loading at the endocortical surface needs further investigation, as it is not caused by higher mechanical strains at this surface, as shown by FE analysis, and might be related to regional differences in osteocyte densities (strain sensors) or connectivity of the osteocyte network. Recent advances in visualization and quantification methods of the osteocyte lacunar canilicular network^37^,^38^ will allow for further investigation of this hypothesis. Additionally, a greater amount of vascularity or the presence of the bone marrow, which has been speculated to play a role in the amplification of mechanical strain may also be contributing to the greater mechanoresponse at the endocortical surface.

Erosion processes were only mechano-responsive on the endocortical surface, since any resorption was hardly detectable for both the control and the loaded mice on the periosteal surface. As a result, adaption was more pronounced on the endocortical than on the periosteal surface, as loading triggered not only greater bone formation on the endocortical compared to periosteal surface, but loading also hindered endocortical bone resorption. On the periosteal surface, only formation processes were enhanced by loading (Video 4,5,6). These regional variations in adaptation do not reflect differences in the local mechanical environment, as the local strain levels are higher at the periosteal surface, due to the bowing of the tibia. Periosteal adaptation to changed loading conditions seems to function only to increase the outwards displacement of bone at levels of high strain via formation processes, but not to increase resorption at levels of low strain, at e.g. the neutral axis of bending, as observed on the endocortical surface. In general, endocortical bone responds to local strain levels by increased formation at high local strains and decreased resorption at low local strains.

Looking at the spatial distribution of remodeling events, the two main features that are highly visible in the loaded cortex of adult mice are that formation and resorption occur in well-separated areas of the endocortical and periosteal surfaces, and that these regions of bone (re)modeling are extended parallel to the axis of the long bone, giving them a striped-like appearance, which is in line with the mechanical environment caused by the bending moment at the bow-shaped mid-diaphysis. Formation occurs at sites of tension (anterior-medial region) and compression (posterior-lateral region), whereas close to the neutral axis resorption cavities are located. This effect was, to a lesser extent, also observed in the young mice, in which formation dominated, and in elderly mice, in which resorption processes dominated. This is in line with previous studies showing bone formation in regions of high strain and resorption in regions of relatively low strain^39^.

With aging the ability of the bone to adapt decreases. Although, both surfaces maintain the ability to increase bone formation in response to loading, the endocortical and periosteal formation response to loading occurred to a lesser degree in older mice. However, this decrease with age is different for the two surfaces. Whereas at the endocortical surface, adult mice show a clearly enhanced adaptive behavior compared to elderly animals, this difference does not exist on the periosteal surface. The response to mechanical stimulation at the periosteal surface is indistinguishable in adult and elderly animals. These data suggest that increased endocortical resorption in the elderly is likely not caused by an adaptation to reduced activity or muscle mass, as suggested by others^40^,^41^, since resorption processes are not mechano-responsive at this surface in the elderly. Furthermore, as the ability to form new bone declines with aging, resorption outpaces formation in the elderly, so that the remodeling cycles are not completed. It is possible, that increased micro-damage in the aged skeleton^42^,^43^ may trigger resorption at the endocortical surface. However, it is not clear why this should be linked solely to the endocortical surface. Studies investigating the distribution of micro-damage in cortical bone may give further insights.

In the elderly mice, endocortical bone formed in response to loading cannot counterbalance the massive endocortical bone loss, since resorption is not mechano-responsive, as the amount of resorbed bone is the same in the loaded and control limbs in the elderly. Adaptation and compensation for this endocortical bone loss seems to be only possible by increasing periosteal formation, which could contribute to the observed periosteal expansion with aging. But also periosteal adaptive formation is not able to increase enough to compensate for the elevated endocortical resorption, leading to a failure in cortical adaptation processes with aging, which results in bone and strength loss reported before by others^44^. We conclude that mechano-responsiveness and changes in the mechano-responsiveness with age depend on the skeletal site, and can be already different in “neighboring” locations in the same bone. Similar regional and age-related differences in mechano-responsiveness may also contribute to compromised cortical integrity in humans with aging, as similar age-related structural changes are also observed in human long bones^45^,^46^,^47^.

In reporting our results, we deliberately avoided the term mechano-sensitivity and used instead mechano-responsiveness to emphasize that in the experiments, the response of bone in the form of a change in bone volume is assessed. The nature of the mechanical stimulus can only be speculated. In the *in vivo* loading experiment, cyclic loading was performed since it is known that changes in loading rather than static loading influences bone remodeling^48^. With commonly used loading protocols, strain rates and strains are proportional to each other (see appendix of^49^), but strains are easier accessible by a FE analysis. Therefore, for the interpretation of our data we used a stimulus related to the local strain, specifically, the local principal strain, which is easy to report as a scalar value. Considering the regional differences of mechano-responsiveness as shown in this study, it can be hypothesized that a reason for these differences could be that the magnitude required or even the nature of the mechanical stimulus needed to trigger adaptation is not necessarily the same in all skeletal sites.

Skeletal aging in mice differs to that in humans^36^ and thus, translation of these results to human bone behavior requires further investigation. Since age-related cortical bone loss in humans resembles to a certain extend our findings in mice^5^,^6^,^31^, it can be speculated that also in humans mechano-responsiveness depends even in the same bone on the skeletal location and could play a role in human cortical thinning in long bones. One limitation of our study is the spatial resolution of the microCT equipment (10.5 μm), which has the greatest influence on the detection of the mineralized thickness (MTh) and the eroded depth (ED)^23^,^24^. However, good agreement with results from histomorphometry demonstrates successful geometric registration of the images^24^. Precision errors (PE_SD_)of the bone volume (0.001), of the endocortical (0.015) and the periosteal bone surface (0.022) determined in a previous *ex vivo* study are more than n order of a magnitude smaller than the mean value of these formation and resorption indices^23^. Therefore, we believe, that the method gives meaningful results. The polychromaticity of the x-ray beam is another potential limitation, since beam hardening effects may occur^50^. We minimized this effect by (1) filtering during scans, (2) a correction algorithm provided by the manufacturer of the scanner and (3) an always similar positioning of the limbs during scanning in a custom-made mouse bed. Another potential limitation is the scan interval chosen. We chose to perform multiple scans at 5-day intervals. The 5-day interval ensured that sufficient mutual information was present between scans to accurately register the images. In addition, having several consecutive scans allows us to choose the best time interval (out of 5 days, 10 days, 15 days) for the analysis. We chose a time interval of 15 days for the thickness and surface area measurements and a five day interval for the volumetric measures. Furthermore, it allows quantifying the time course of the response. Averaging over all animals, our data shows that the response is almost linear. Locally higher strain rates might occur. By doing a strain-matched experiment, we ensured that the strain rate at the strain gage location was the same across the different age groups. However, due to the shape of the bone, axial compression results in a highly heterogeneous strain environment independently of age and therefore, it would be impossible to induce the same strain rate in all the regions within the bone and to match that across the different ages.

In conclusion our key findings are: 1) Both cortical bone surfaces respond to mechanical stimulation in the expected way, by an increase in formation. Concerning resorption, the situation is asymmetric with a decrease in resorption on the endocortical surface in response to loading, while virtually no resorption was detected on the periosteal surface in both control and loaded animals during the time interval of monitoring. 2) The endocortical surface was found to be more mechano-responsive than the periosteal surface, as endocortical strains are lower, but this region still responds more. In each age group, formation parameters are more strongly increased due to loading on the endocortical compared to the periosteal surface. 3) Mechano-responsiveness declines with increasing animal age on both surfaces and the response to loading on the periosteal surface of adult animals is already as low as the response on the endocortical surface for the elderly animals. The decline of mechano-responsiveness with age on the two cortical surfaces can be summarized as a decline that is already more advanced at the periosteal surface and, therefore, the periosteal surface is “older” in terms of mechano-responsiveness than the endocortical surface.

## Additional Information

**How to cite this article**: Birkhold, A. I. *et al*. The Periosteal Bone Surface is Less Mechano-Responsive than the Endocortical. *Sci. Rep.* **6**, 23480; doi: 10.1038/srep23480 (2016).

## Supplementary Material

Supplementary Information

Supplementary Video 1

Supplementary Video 2

Supplementary Video 3

Supplementary Video 4

Supplementary Video 5

Supplementary Video 6
