# Supplementary material for: The Periosteal Bone Surface is Less Mechano-Responsive than the Endocortical
Source: Sci Rep. 2016 Mar 23;6:23480. doi: 10.1038/srep23480 (PMC4804282; doi:10.1038/srep23480)
Supplement: Supplementary Information [file srep23480-s1.doc]

**The Periosteal Bone Surface is Less Mechano-Responsive than the Endocortical**

Annette I. Birkhold, Hajar Razi, Georg N. Duda, Richard Weinkamer, Sara Checa, Bettina M. Willie

***Supplementary Table S1: 3D dynamic (re)modeling parameters on endocortical surface of control and loaded bones. * indicates a significant difference between loaded and control bones.***

| **Age** |  |  | **young** |  |  | **adult** |  |  | **elderly** |  |
| --- | --- | --- | --- | --- | --- | --- | --- | --- | --- | --- |
| **Day** |  | **5** | **10** | **15** | **5** | **10** | **15** | **5** | **10** | **15** |
| **Control** |  |  |  |  |  |  |  |  |  |  |
| **MV/BV** | *[µm³/µm³*10*-³*]* | 3.04±1.31 | 7.85±7.31 | 14.11±9.80 | 0.87±0.48 | 2.10±1.32 | 2.18±1.23 | 1.95±3.98 | 3.90±3.07 | 5.12±3.50 |
| **MS/BS** | *[µm²/µm²*10*-²*]* | 3.60±1.31 | 9.15±7.89 | 16.11±9.37 | 1.38±0.84 | 1.34±0.85 | 3.25±1.78 | 2.22±4.57 | 4.04±2.90 | 5.12±3.81 |
| **MTh** | *[µm]* | 15.76±0.01 | 15.77±0.02 | 15.84±0.16 | 15.77±0.03 | 15.82±0.17 | 15.88±0.16 | 15.92±0.35 | 15.93±0.33 | 16.13±0.50 |
| **3D BFR** | *[µm³/day*10⁴]* |  |  | 38.41±23.79 |  |  | 8.46±4.89 |  |  | 18.01±12.45 |
| **3D MAR** | *[µm/day]* |  |  | 1.06±0.01 |  |  | 1.06±0.01 |  |  | 1.08±0.03 |
| **EV/BV** | *[µm³/µm³*10*-³*]* | 1.63±0.98 | 1.41±1.30 | 1.53±1.5 | 6.53±6.56 | 10.70±10.70 | 32.46±13.79 | 10.20±11.24 | 26.50±16.53 | 48.31±17.62 |
| **ES/BS** | *[µm²/µm²*10*-²*]* | 1.98±1.21 | 1.77±1.62 | 1.82±1.67 | 8.65±8.02 | 20.48±11.78 | 34.56±10.64 | 10.62±11.01 | 22.76±12.19 | 38.48±12.77 |
| **ED** | *[µm]* | 15.76±0.02 | 15.75±0.01 | 15.84±0.21 | 16.03±0.41 | 16.98±0.77 | 19.30±2.10 | 16.00±0.37 | 18.04±1.65 | 20.18±2.16 |
| **3D BRR** | *[µm³/day*10⁴]* |  |  | 4.76±5.28 |  |  | 125.58±54.21 |  |  | 169.65±65.85 |
| **3D MRR** | *[µm/day]* |  |  | 1.06±0.01 |  |  | 1.29±0.14 |  |  | 1.35±0.14 |
| **Loaded** |  |  |  |  |  |  |  |  |  |  |
| **MV/BV** | *[µm³/µm³*10-³]* | 9.43±1.56* | 42.9±11.64* | 77.14±30.88* | 3.15±2.63* | 8.37±4.55* | 26.19±16.45* | 4.05±3.25 | 4.04±2.45 | 11.15±6.01* |
| **MS/BS** | *[µm²/µm²*10-²]* | 10.77±1.44* | 41.65±8.4* | 56.64±20.90* | 4.88±4.05* | 1.24±6.17* | 30.57±14.79* | 5.19±4.22* | 5.22±3.34 | 12.77±6.30* |
| **MTh** | *[µm]* | 15.79±0.03* | 16.92±0.67* | 21.04±2.19* | 15.78±0.06 | 15.88±0.14 | 18.10±2.70* | 15.80±0.08 | 15.79±0.06 | 16.13±0.32 |
| **3D BFR** | *[µm³/day*10⁴]* |  |  | 210.0±82.72 |  |  | 98.25±58.96* |  |  | 40.05±21.71* |
| **3D MAR** | *[µm/day]* |  |  | 1.40±0.14* |  |  | 1.21±0.18* |  |  | 1.08±0.02 |
| **EV/BV** | *[µm³/µm³*10-³]* | 0.81±0.52 | 0.48±0.53 | 1.86±4.08 | 1.28±1.81* | 6.64±3.96* | 16.19±6.04* | 4.92±2.25 | 24.28±6.32 | 39.06±1.36 |
| **ES/BS** | *[µm²/µm²*10-²]* | 0.92±0.53* | 0.52±0.52 | 2.10±4.62 | 1.86±2.53* | 9.90±5.87* | 16.51±6.27* | 5.82±2.63 | 22.53±5.78 | 31.58±9.76 |
| **ED** | *[µm]* | 15.75±0.01 | 15.82±0.15 | 15.80±0.11 | 15.78±0.02 | 16.96±1.07 | 21.40±5.38 | 15.96±0.22 | 18.21±1.50 | 21.52±3.21 |
| **3D BRR** | *[µm³/day*10⁴]* |  |  | 0.002±0 |  |  | 62.61±23.97* |  |  | 141.91±53.12 |
| **3D MRR** | *[µm/day]* |  |  | 1.05±0.01 |  |  | 1.43±0.36 |  |  | 1.43±0.21 |

***Supplementary Table S2***: 3D dynamic (re)modeling parameters on periosteal surface of control and loaded bones. * indicates a significant difference between loaded and control bones.

| **Age** |  |  | **young** |  |  | **adult** |  |  | **elderly** |  |
| --- | --- | --- | --- | --- | --- | --- | --- | --- | --- | --- |
| **Day** |  | **5** | **10** | **15** | **5** | **10** | **15** | **5** | **10** | **15** |
| **Control** |  |  |  |  |  |  |  |  |  |  |
| **MV/BV** | *[µm³/µm³*10*-³*]* | 2.74±1.44 | 4.14±3.87 | 8.70±6.85 | 0.83±1.03 | 0.86±0.72 | 1.58±2.35 | 1.14±2.03 | 0.71±0.85 | 1.13±1.65 |
| **MS/BS** | *[µm²/µm²*10*-²*]* | 2.55±1.31 | 5.20±4.65 | 8.29±6.50 | 0.95±1.22 | 0.99±0.85 | 1.79±2.69 | 1.21±2.18 | 0.71±0.86 | 1.10±1.58 |
| **MTh** | *[µm]* | 15.75±0.0 | 15.77±0.05 | 15.77±0.05 | 15.75±0.0 | 15.75±0.0 | 15.76±0.05 | 15.75±0.0 | 15.75±0.0 | 15.75±0.0 |
| **3D BFR** | *[µm³/day*10⁴]* |  |  | 24.27±19.48 |  |  | 6.05±8.93 |  |  | 4.01±5.80 |
| **3D MAR** | *[µm/day]* |  |  | 1.05±0.0 |  |  | 1.05±0.0 |  |  | 1.05±0.0 |
| **EV/BV** | *[µm³/µm³*10*-³*]* | 0.86±0.34 | 1.11±0.88 | 0.96±0.82 | 0.70±0.83 | 1.18±0.93 | 1.73±1.96 | 0.46±0.70 | 1.44±2.76 | 2.13±3.92 |
| **ES/BS** | *[µm²/µm²*10*-²*]* | 0.81±0.35 | 1.06±0.83 | 0.90±0.73 | 0.77±0.92 | 1.33±1.06 | 1.99±2.32 | 0.48±0.77 | 1.41±2.63 | 2.22±4.23 |
| **ED** | *[µm]* | 15.75±0.0 | 15.77±0.07 | 15.75±0.0 | 15.75±0.0 | 15.75±0.0 | 15.76±0.01 | 15.75±0.0 | 15.75±0.0 | 15.83±0.24 |
| **3D BRR** | *[µm³/day*10⁴]* |  |  | 2.63±2.0 |  |  | 6.89±8.21 |  |  | 8.02±15.67 |
| **3D MRR** | *[µm/day]* |  |  | 1.05±0.0 |  |  | 1.05±0.0 |  |  | 1.06±0.02 |
| **Loaded** |  |  |  |  |  |  |  |  |  |  |
| **MV/BV** | *[µm³/µm³*10-³]* | 6.75±2.52* | 18.85±6.08* | 53.07±6.17* | 15.33±1.92 | 5.13±3.59* | 8.31±5.28* | 3.54±2.86 | 1.77±2.15 | 6.93±5.12* |
| **MS/BS** | *[µm²/µm²*10-²]* | 6.22±2.16* | 17.90±6.12* | 51.38±6.63* | 1.68±2.09 | 5.96±4.42* | 9.38±5.99* | 3.58±2.87 | 1.87±2.33 | 7.19±5.38* |
| **MTh** | *[µm]* | 15.76±0.01 | 15.81±0.07 | 17.5±2.95 | 15.75±0.00 | 15.75±0.01 | 15.80±0.11 | 15.77± | 15.75±0.00 | 15.75±0.01* |
| **3D BFR** | *[µm³/day*10⁴]* |  |  | 148±27.18* |  |  | 31.80±20.40* |  |  | 25.63±19.73 |
| **3D MAR** | *[µm/day]* |  |  | 1.17±0.20 |  |  | 1.05±0.01 |  |  | 1.05±0.00 |
| **EV/BV** | *[µm³/µm³*10-³]* | 0.61±0.46 | 0.23±0.25 | 0.12±0.19 | 0.99±1.12 | 0.85±1.76 | 0.49±0.53 | 0.77±0.98 | 1.03±1.20 | 0.73±1.32 |
| **ES/BS** | *[µm²/µm²*10-²]* | 0.56±0.41 | 2.09±0.22 | 1.21±0.19 | 1.10±1.26 | 0.91±1.82 | 0.56±0.61 | 0.80±1.05 | 1.04±1.22 | 0.72±1.28 |
| **ED** | *[µm]* | 15.76±0.03 | 15.75±0.00 | 15.75±0.00 | 15.75±0.00 | 15.75±0.00 | 15.75±0.01 | 15.75±0.01 | 15.78±0.10 | 15.75±0.01 |
| **3D BRR** | *[µm³/day*10⁴]* |  |  | 0.37±0.61 |  |  | 1.88±2.02 |  |  | 2.60±4.77 |
| **3D MRR** | *[µm/day]* |  |  | 1.05±0.00 |  |  | 1.05±0.00 |  |  | 1.05±0.00 |

***Video Legends:***

*Video 1: Visualization of periosteal (re)modeling in a control tibia from a young mouse.*

*Video 2: Visualization of periosteal (re)modeling in a control tibia from an adult mouse.*

*Video 3: Visualization of periosteal (re)modeling in a control tibia from an elderly mouse.*

*Video 4: Visualization of periosteal (re)modeling in a loaded tibia from a young mouse.*

*Video 5: Visualization of periosteal (re)modeling in a loaded tibia from an adult mouse.*

*Video 6: Visualization of periosteal (re)modeling in a loaded tibia from an elderly mouse.*
